# Risk factors for metastasis and poor prognosis of Ewing sarcoma: a population based study

**DOI:** 10.1186/s13018-020-01607-8

**Published:** 2020-03-04

**Authors:** Jiaqi Shi, Jianing Yang, Xin Ma, Xu Wang

**Affiliations:** 1 Department of Orthopedics, Huashan Hospital, Fudan University, 12, Wulumuqi Rd., Jing’an District, Shanghai, China; 2 Department of Radiotherapy, Oncology Hospital, Fudan University, Shanghai, China

**Keywords:** Ewing sarcoma, Metastasis, Nomograms, Prognosis, Risk factors

## Abstract

**Background:**

This study is to determine the risk factors for metastasis of Ewing sarcoma (ES) patients in SEER database. Then explore clinicopathological factors associated with poor prognosis. Furthermore, develop the nomogram to predict the probability of overall survival and cancer-specific survival

**Methods:**

Thus, we collected clinicopathological data of ES patients in SEER database, and then used chi-square test and logistic regression to determine risk factors associated to metastasis. We also did survival analysis including Kaplan-Meier curve and Cox proportional hazard model to explore the risk factors associated to overall survival and cancer-specific survival, and then developed the nomogram to visualize and quantify the probability of survival.

**Results:**

After statistics, we find that patients with older ages (11–20 years old: OR = 1.517, 95% confidence interval [CI] 1.033–2.228, *p* = 0.034; 21–30 years old: OR = 1.659. 95%CI 1.054–2.610, *p* = 0.029), larger tumor size (> 8 cm: OR = 1.914, 95%CI 1.251–2.928, *p* = 0.003), and pelvic lesions (OR = 2.492, 95%CI 1.829–3.395, *p* < 0.001) had a higher risk of metastasis. ROC curves showed higher AUC (0.65) of combined model which incorporate these three factors to predict the presence of metastasis at diagnosis. In survival analysis, patients with older ages (11–20 years: HR = 1.549, 95%CI 1.144–2.099, *p* = 0.005; 21–30 years: HR = 1.808, 95%CI 1.278–2.556, *p* = 0.001; 31–49 years: HR = 3.481, 95%CI 2.379–5.094, *p* < 0.001; ≥ 50 years: HR = 4.307, 95%CI 2.648–7.006, *p* < 0.001) , larger tumor size (5–8 cm: HR = 1.386, 95%CI 1.005–1.991, *p* = 0.046; > 8 cm: HR = 1.877, 95%CI 1.376–2.561, *p* < 0.001), black race (HR = 2.104, 95%CI 1.296–3.416, *p* = 0.003), and wider extension (regional: HR = 1.373, 95%CI 1.033–1.823, *p* = 0.029; metastatic: HR = 3.259, 95%CI 2.425–4.379, *p* < 0.001) were associated with worse prognosis. Chemotherapy was associated with better prognosis (HR = 0.466, 95%CI 0.290–0.685, *p* < 0.001). The nomogram which developed by training set and aimed to predict OS and CSS showed good consistency with actual observed outcomes internally and externally.

**Conclusion:**

In conclusion, tumor size and primary site were associated with distant metastasis at diagnosis. Age, tumor size, primary site, tumor extent, and chemotherapy were associated with overall survival and cancer-specific survival. Nomogram could predict the probability of OS and CSS and showed good consistency with actual observed outcomes internally and externally.

## Introduction

Ewing sarcoma (ES) is the second most common primary malignant bone tumor in children and adolescents—second only to osteosarcoma [1]. The highest incidence is in the second decade of life with approximately 9 to 10 cases per million per year seen in patients aged 10–19 years [2]. ES arises mostly in the bone and skeletal ES most frequently involves the diaphysis or metadiaphyseal region of the long bones [3]. The pelvis, ribs, and spine are also commonly involved [4].

Previous studies have discussed prognostic factors of Ewing sarcoma. These studies have shown that advanced age, large tumor volume, axial tumor location, as well as metastatic disease at presentation are independent risk factors for poor prognosis [5–12]. Of these, metastasis at diagnosis appears to be the most common prognostic risk factor. However, risk factors for metastasis at diagnosis of ES remain dismal.

**Fig. 1:**
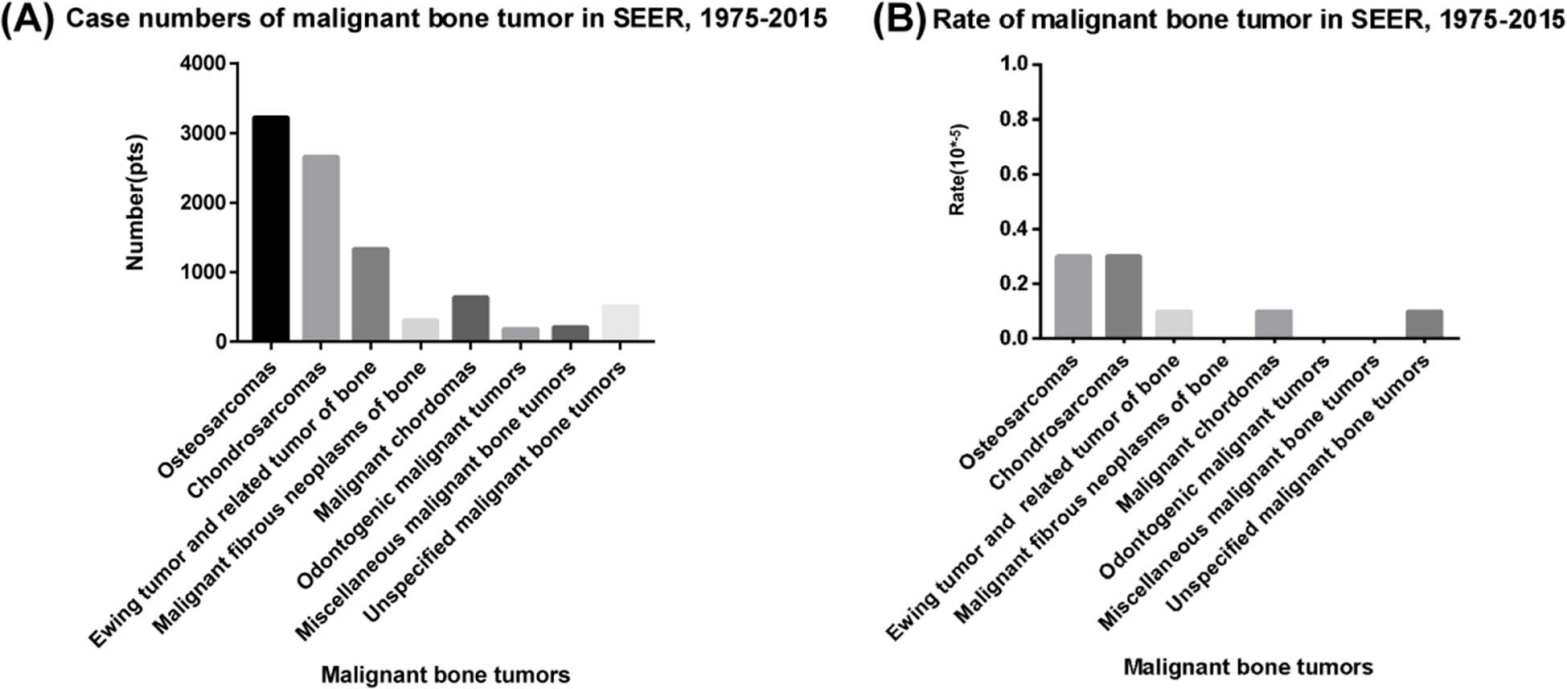
The incidence of malignant bone tumors in the SEER database from 1975 to 2015 **a** number, **b** rate (10^−5^)

There is a very low incidence of ES [2], and it is quite challenging to enroll a sufficient number of ES patients into a study cohort. Thus, we used the Surveillance, Epidemiology, and End Results (SEER) database to enroll a sufficient number of cases. The SEER database consists of 18 cancer registries and covers approximately 30% of the total US population. This analysis showed the incidence of malignant bone tumors from 1975 to 2015; the total population was 1,069,346,173. The total number of malignant bone tumor patients was 9,054, which is 0.9% of all malignant tumor patients (Fig. 1a, b).

In our study, based on the data of ES patients from SEER database, we identified risk factors for metastasis, as well as prognostic factors for overall survival (OS) and cancer-specific survival (CSS). Then we developed the nomogram to predict the probability of OS and CSS in ES patients.

## Materials and methods

### Clinical data and selection criteria

We used the SEER data that contains the epidemiological information of 18 cancer registries in the USA. SEER*Stat (Version 8.3.5) is produced by the Surveillance Research Program of the Division of Cancer Control and Population Sciences, National Cancer Institute to extract the data from SEER database. In the software, we chose database called “Incidence=SEER 18 Regs Custom Data (with additional treatment fields), Nov. 2017 Sub (1973–2015 varying)” which contained treatment information of ES patients, including radiation therapy and chemotherapy.

The inclusion criteria were (1) patients diagnosed with Ewing tumor of the bones and joints according to the Site and Morphology AYA site recode/WHO2008; (2) patients diagnosed between 1983 and 2015 because the clinicopathologic data before 1983 was mostly incomplete, and some variables such as tumor size were not clear; (3) ES of the bones and joints was the first and only primary malignant tumor; (4) complete clinical data, including age at diagnosis, gender, race, primary site, tumor size, tumor extension, the state of distant metastasis, cancer-directed surgery, and treatments (radiation and chemotherapy) performed or not; (5) diagnosis was acquired from a living patient—not autopsy or death certificate; (6) complete follow-up data and known follow-up time; and (7) known cause of death and survival time after diagnosis.

The exclusion criteria were (1) ES of the other extra-bone sites or the soft tissues except the bones, such as the abdominal and pelvic cavity; (2) histologically classified as pNET and Askin sarcoma; (3) patients had other concurrent malignant tumors or Ewing sarcoma was not the first malignant tumor; and (4) incomplete clinicopathological and survival data.

### Risk factors of metastasis

We explored the risk factors of distant metastasis at diagnosis of ES patients. The state of metastasis showed in the tumor extent variable. Tumor extent was divided into three categories: localized (confined to the periosteum) , regional (extended beyond the periosteum to the surrounding tissue but without distant metastasis), and metastatic (with distant metastasis at diagnosis). The localized and regional were classified into non-metastatic group, and the metastatic was classified into metastatic group.

We chose five clinicopathologic variables as alternative risk factors: age (age at diagnosis), gender, race, primary site, and tumor size. Age was categorized into five groups, which were less than 10 years old, between 11 to 20 years old, between 21 to 30 years old, between 31 to 49 years old, and over 50 years old. Gender included male and female, and race included white, black, and others (Native American/Alaska Native, Asian/Pacific Islander). Primary sites were categorized into five groups: the limb (including the long and short bones of the upper and lower extremities), cranial, spine, thoracic, and pelvic. Tumor size was divided into three groups: < 5 cm, 5–8 cm, and > 8 cm. Univariate logistic regression was used to select variables as possible risk factors associated with metastasis at diagnosis, and *p* < 0.05 was considered statistically significant. Then multivariate logistic regression was applied to determine the risk factors selected in the univariate regression.

ROC curve (receiver operating characteristic curve) showed the prediction power of each risk factor and five-factor combined model, and AUC (area under curve) value was also listed. Higher AUC presented higher prediction power.

Besides, we also showed the additional treatment information, including cancer-directed surgery, radiation, and chemotherapy. Together with the five alternative risk factors, chi-squared test was used to compare the differences of these clinicopathological factors between non-metastatic and metastatic groups.

### Prognostic analysis

The overall survival (OS) was an endpoint of interest. OS was defined as the time from diagnosis of death from all possible causes. Patients who were alive at the time of the last follow-up were considered censored data. We also considered cancer-specific survival (CSS) as the other endpoint of interest and defined it as the time from diagnosis to death from cancer. Patients who were alive or dead of other causes except ES at the time of the last follow-up were considered censored data.

In prognostic analysis, survival curves for each variable were estimated by Kaplan-Meier method, and the significance of the differences between survival curves was determined by log-rank test. We also used Cox regression to do survival analysis. Univariate Cox regression was used to select variables as possible risk factors related to OS and CSS. Then multivariate Cox regression was applied to determine the risk factors for OS and CSS selected in the univariate Cox regression.

### Nomogram development and validation

We aimed to construct the nomogram to predict the probability of OS and CSS in ES patients. All patients were randomly assigned into training set and validation set by the ratio 1:1. The nomogram was developed by data of the training set. Internal validation (training set) and external validation (validation set) were conducted with 1000 bootstrap resamples to prevent overfitting. The predictive power of the nomogram was assessed by Harrell’s concordance index (C-index). The value of C-index should range from 0.5 to 1.0, and 0.5 indicates random chance and 1.0 indicates a perfectly corrected discrimination. We also used calibration plots to compare nomogram predicting results with actual observed survival outcomes both internally and externally.

### Statistical analysis

#### Risk factors of metastasis

Univariate logistic regression was used to select variables as possible risk factors associated with metastasis at diagnosis. Then multivariate logistic regression was applied to determine the risk factors selected in the univariate regression. ROC curve (receiver operating characteristic curve) showed the prediction power of each risk factor and five-factor combined model, and AUC (area under curve) value was also listed. Chi-squared test was used to compare the differences of these clinicopathological factors between non-metastatic and metastatic groups. Chi-squared test as well as univariate and multivariate logistic regression was performed with SPSS Version 23.0 (IBM Corporation), and *p* < 0.05 was considered statistically significant.

#### Survival analysis

Survival curves for each variable were estimated by Kaplan-Meier method, and the significance of the differences between survival curves was determined by log-rank test. Univariate Cox regression was used to select variables as possible risk factors related to OS and CSS. Then multivariate Cox regression was applied to determine the risk factors for OS and CSS selected in the univariate Cox regression. Log-rank test and Cox regression were also performed by SPSS Version 23.0 (IBM Corporation). All *p* values were two-sided, and we assumed that a probability less than 0.05 was statistically significant.

#### Nomogram development and validation

The development and validation of the nomogram were performed by R version 3.5.1 (http://www.r-project.org/) with rms [13] and cmprsk [14] packages.

## Results

### Patient baseline characteristics

**Fig. 2:**
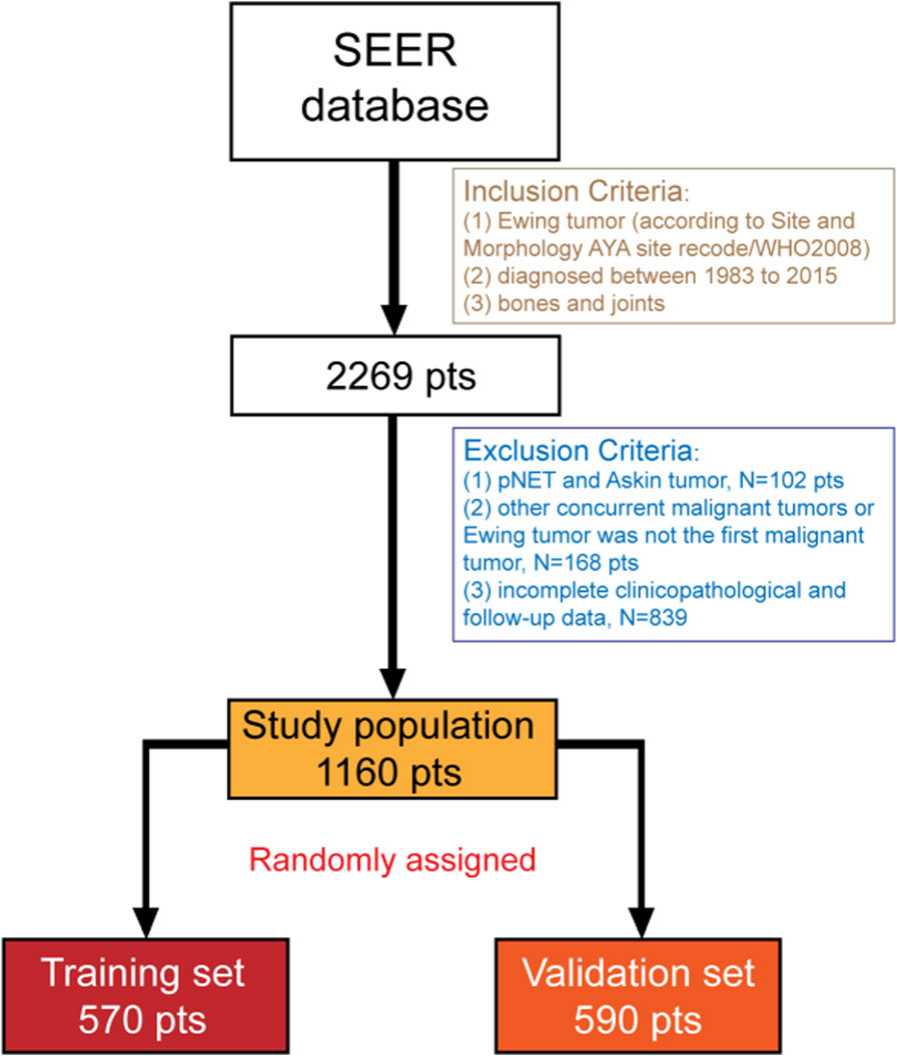
The diagram showed the enrolling process. Based on the inclusion and exclusion criteria, finally 1160 patients from SEER database were included in this study. After random assignment, 570 and 590 patients were assigned into the training set and the validation set respectively

Based on the SEER database, we collected the data of total patients diagnosed with ES from 1983 to 2015. In the Incidence=SEER 18 Regs Custom Data(with additional treatment fields), Nov. 2017 Sub (1973–2015 varying) database, the total number of patients who were diagnosed with Ewing sarcoma of the bones and joints was 2269. After the screening of inclusion and exclusion criteria, 1160 patients were finally included in our study (Fig. 2).

Table 1 shows the patient baseline demographic and clinicopathologic characteristics. For categorical variables, we showed the frequency and percentage. Most were young patients, and 813 (70.1%) patients were aged below 20 years. There were 733 (63.2%) male patients and 427 (36.8%) female patients, and the ratio was approximately 2:1, which probably indicated that boys and men are slightly more affected than girls and women. The demographic baseline data showed above was mostly consistent with the previous epidemiology investigation of ES [15]. Of the 1160 patients, 1041 (89.7%) were white. The most common involved primary site was the limb (*N* = 519, 44.7%) followed by the pelvis (sacrum and coccyx, *N* = 314, 27.1%). As for tumor extent, less than 1/3(311, 26.8%) were localized, and nearly 1/2 (533, 45.9%) were regionally extended, and 316 (27.2%) patients were detected distant metastatic lesions at diagnosis. The traditional treatments for ES were surgery, radiation, and chemotherapy. Cancer-directed surgery was performed in 770 (66.4%) patients. 576 (49.7%) received radiation, and nearly all patients (1120, 96.6%) received chemotherapy.

**Table 1:** Baseline demographics and clinicopathological characteristics of all patients (metastatic and non-metastatic groups)

Characteristic	All patients(*N* = 1160, 100%) Number (%)	Non-metastatic(*N* = 844, 72.8%) Number (%)	Metastatic(*N* = 316, 27.2%) Number (%)	*p* value
Age (years)				0.011*
≤ 10	242 (20.9%)	197 (23.3%)	45 (14.2%)	
11–20	571 (49.2%)	402 (47.6%)	169 (53.5%)	
21–30	206 (17.8%)	142 (16.8%)	64 (20.3%)	
31-49	101 (8.7%)	76 (9.0%)	25 (7.9%)	
≥ 50	40 (3.4%)	27 (3.2%)	13 (4.1%)	
Gender				0.926
Male	733 (63.2%)	534 (63.3%)	199 (63.0%)	
Female	427 (36.8%)	310 (36.7%)	117 (37.0%)	
Race				0.749
White	1041 (89.7%)	759 (89.9%)	282 (89.2%)	
Black	30 (2.6%)	20 (2.4%)	10 (3.2%)	
Other*	89 (7.7%)	65 (7.7%)	24 (7.6%)	
Primary site				< 0.001***
Limb	519 (44.7%)	405 (48.0%)	114 (36.1%)	
Cranial	66 (5.7%)	60 (7.1%)	6 (1.9%)	
Spine	88 (7.6%)	71 (8.4%)	17 (5.4%)	
Thoracic	173 (14.9%)	129 (15.3%)	44 (13.9%)	
Pelvic	314 (27.1%)	179 (21.2%)	135 (42.7%)	
Tumor size (cm)				< 0.001***
< 5	221 (19.1%)	185 (21.9%)	36 (11.4%)	
5–8	371 (32.0%)	283 (33.5%)	88 (27.8%)	
> 8	568 (49.0%)	376 (44.5%)	192 (60.8%)	
Tumor extent				-
Localized	311 (26.8%)	311 (36.8%)	0 (0.0%)	
Regional	533 (45.9%)	533 (63.2%)	0 (0.0%)	
Metastatic	316 (27.2%)	0 (0.0%)	316 (100.0%)	
Cancer-directed surgery				< 0.001***
No	390 (33.6%)	213 (25.2%)	177 (54.0%)	
Yes	770 (66.4%)	631 (74.8%)	139 (44.0%)	
Radiation				< 0.001***
No	584 (50.3%)	468 (55.5%)	116 (36.7%)	
Yes	576 (49.7%)	376 (44.5%)	200 (63.3%)	
Chemotherapy				0.49
No	40 (3.4%)	31 (3.7%)	9 (2.8%)	
Yes	1120 (96.6%)	813 (96.3%)	307 (97.2%)	

*p* value < 0.05*, < 0.001***

*Including Native American/Alaska Native, Asian/Pacific Islander

### Risk factors for metastasis in Ewing sarcoma patients

All patients were divided into two groups according to the state of metastasis at diagnosis. Table 1 also shows the results of chi-squared test between non-metastatic and metastatic groups. The results indicated that the metastatic group showed older ages (*p* = 0.011), more pelvic tumors (*p* < 0.001) and larger tumor size (*p* < 0.001). These two groups tended to receive different treatments after diagnosis. The proportion of cancer-directed surgery in non-metastatic group was higher (*p* < 0.001), but the proportion of radiation was higher in the metastasis group (*p* < 0.001). Because nearly all the patients received chemotherapy, there was no statistical significance between two groups.

The results of logistic regression (Table 2) indicated that age, primary site, and tumor size were associated with distant metastasis at diagnosis (all *p* < 0.05). In detail, the OR (odds ratio) value showed the relative risk of the presence of metastasis at diagnosis. In multivariate logistic regression, older age had a higher risk relative to aged below 10 years old, which indicated that older patients were more likely to be metastatic at diagnosis (11–20 years old: OR = 1.517, 95% confidence interval [CI] 1.033–2.228, *p* = 0.034; 21–30 years old: OR = 1.659. 95%CI 1.054–2.610, *p* = 0.029). But the groups of 31–49 years old and over 50 years old did not show the same trend(*p* = 0.264 and 0.058), and one of the possible reasons was the smaller numbers of patients in these groups. Besides, patients with larger tumors (> 8 cm: OR = 1.914, 95%CI 1.251–2.928, *p* = 0.003) had a higher risk for the presence of metastasis. Relative to the limb, pelvic lesions had a higher risk for the presence of metastasis (OR = 2.492, 95%CI 1.829–3.395, *p* < 0.001).

**Table 2:** Univariate and multivariate Logistic regression for risk factors of metastasis in Ewing tumor patients

	Univariate		Multivariate	
Variables	OR (95% CI)	*p* value	OR (95% CI)	*p* value
Age (years)				
≤ 10	Reference	0.012*	Reference	0.144
11–20	1.840 (1.271–2.665)	0.001**	1.517 (1.033–2.228)	0.034*
21–30	1.973 (1.273–3.058)	0.002**	1.659 (1.054–2.610)	0.029*
31–49	1.440 (0.826–2.511)	0.199	1.388 (0.780–2.470)	0.264
≥ 50	2.108 (1.009–4.403)	0.047*	2.082 (0.977–4.438)	0.058
Gender				
Male	Reference			
Female	1.013 (0.775–1.324)	0.926		
Race				
White	Reference	0.751		
Black	1.346 (0.622–2.910)	0.451		
Other	0.994 (0.610–1.618)	0.980		
Primary site				
Limb	Reference	< 0.001***	Reference	< 0.001***
Cranial	0.355 (0.150–0.843)	0.019*	0.486 (0.201–1.176)	0.110
Spine	0.851 (0.482–1.502)	0.577	1.033 (0.572–1.865)	0.914
Thoracic	1.212 (0.812–1.808)	0.347	1.245 (0.830–1.867)	0.289
Pelvic	2.679 (1.975–3.635)	< 0.001***	2.492 (1.829–3.395)	< 0.001***
Tumor size (cm)				
< 5	Reference	< 0.001***	Reference	0.005**
5–8	1.598 (1.040–2.456)	0.033*	1.362 (0.871–2.131)	0.176
> 8	2.624 (1.764–3.903)	< 0.001***	1.914 (1.251–2.928)	0.003**

*OR* odds ratio

*p* value < 0.05*, < 0.01**, < 0.001***

**Fig. 3:**
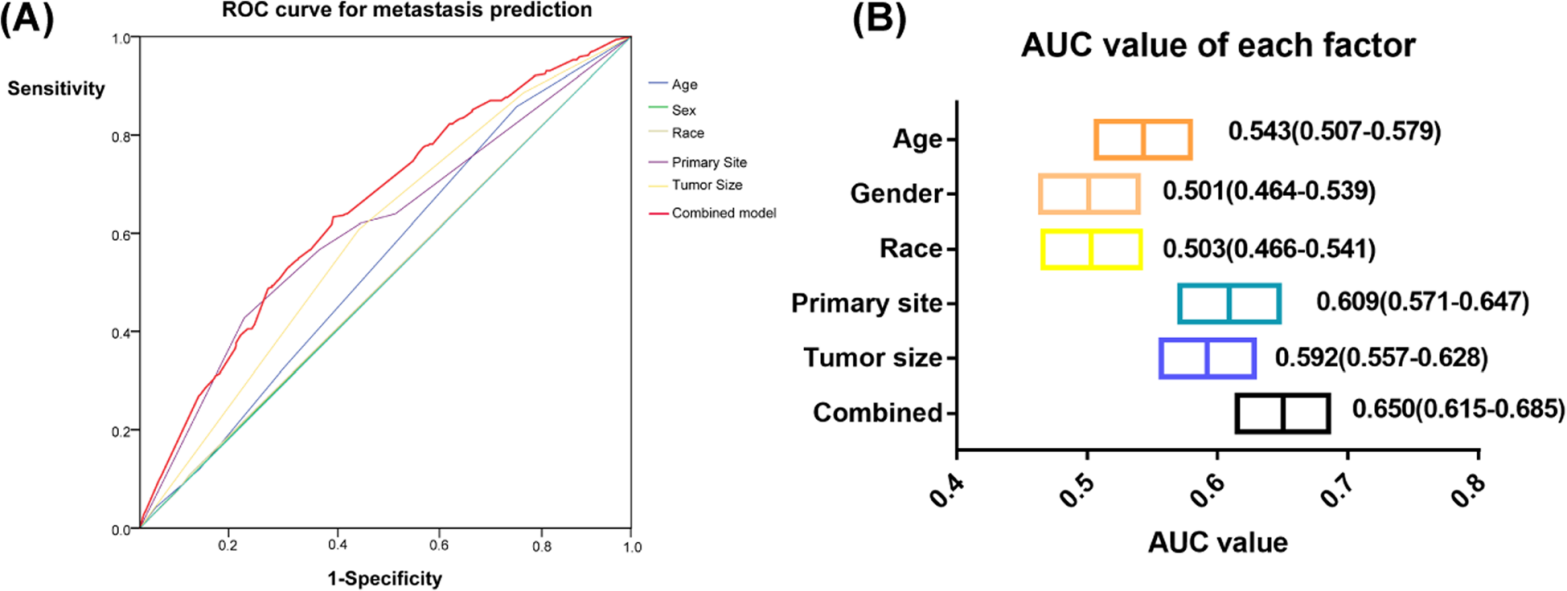
The ROC curve and AUC of age, sex, race, primary site, tumor size, and combined model. **a** ROC curves. **b** AUC value and its 95% confident interval

The ROC curves for age, sex, race, primary site, and tumor size were showed below (Fig. 3). The combined model was the multivariate logistic regression function included age, primary site, and tumor size together. The AUC of the combined model was the highest (AUC 0.650, 95%CI 0.615–0.685), which indicated the combined model showed the highest prediction power of metastasis at diagnosis.

### Prognostic factors for survival in Ewing sarcoma

In the treatment and management of cancer patients, the ultimate goal is to improve patients’ survival. Here, we chose these two prognostic indicators: overall survival (OS) and cancer-specific survival (CSS). We used the univariate Cox regression to analyze whether a variable was associated with survival, and then multivariate analysis was performed to determine whether this variable was an independent prognostic factor. The results of OS and CSS were mostly concordant (Tables 3 and 4). The Cox regression showed that older patients, larger tumor size, black race, wider extension, metastasis at diagnosis, and without chemotherapy were associated with worse prognosis; all of these factors were independent prognostic factors. More specifically, we compared those with limb lesions to patients with pelvic and spine lesions, and the latter had worse survival. Furthermore, we also noticed that cancer-directed surgery was related to longer CSS and OS in univariate analysis, but the effect disappeared in multivariate analysis. Radiation also showed the same effect, and the reason remained unknown.

**Table 3:** Univariate and multivariate Cox regression analysis for overall survival(OS) in Ewing tumor patients

	Univariate		Multivariate	
Variables	HR (95% CI)	*p* value	HR (95% CI)	*p* value
Age (years)				
≤ 10	Reference	< 0.001***	Reference	< 0.001***
11–20	1.873 (1.388–2.528)	< 0.001***	1.549(1.144–2.099)	0.005**
21–30	2.351 (1.674–3.301)	< 0.001***	1.808(1.278–2.556)	0.001**
31–49	3.494 (2.410–5.064)	< 0.001***	3.481(2.379–5.094)	< 0.001***
≥ 50	4.557 (2.831–7.336)	< 0.001***	4.307 (2.648–7.006)	< 0.001***
Gender				
Male	Reference			
Female	0.905 (0.746–1.099)	0.313		
Race				
White	Reference	0.008**	Reference	0.011*
Black	2.120 (1.321–3.403)	0.002**	2.104 (1.296–3.416)	0.003**
Other	1.025 (0.715–1.469)	0.894	1.107 (0.706–1.465)	0.928
Primary site				
Limb	Reference	< 0.001***	Reference	0.004**
Cranial	1.139 (0.730–1.778)	0.566	1.480 (0.922–2.377)	0.104
Spine	1.478 (1.034–2.111)	0.032*	1.458 (0.999–2.129)	0.051
Thoracic	1.062 (0.796–1.418)	0.681	0.909 (0.675–1.226)	0.532
Pelvic	1.855 (1.493–2.305)	< 0.001***	1.462 (1.151–1.858)	0.002**
Tumor size (cm)				
< 5	Reference	< 0.001***	Reference	< 0.001***
5–8	1.395 (1.025–1.898)	0.034*	1.386 (1.005–1.911)	0.046*
> 8	1.985 (1.493–2.640)	< 0.001***	1.877 (1.376–2.561)	< 0.001***
Tumor extent				
Localized	Reference	< 0.001***	Reference	< 0.001***
Regional	1.512(1.148–1.991)	0.003*	1.373 (1.033–1.823)	0.029*
Metastatic	3.820(2.897–5.038)	< 0.001***	3.259 (2.425–4.379)	< 0.001***
Cancer-directed surgery				
No	Reference		Reference	
Yes	0.571 (0.473–0.690)	< 0.001***	0.933 (0.749–1.172)	0.536
Radiation				
No	Reference		Reference	
Yes	1.352 (1.122–1.630)	0.002**	1.065 (0.874–1.299)	0.531
Chemotherapy				
No	Reference		Reference	
Yes	0.426 (0.282–0.642)	< 0.001***	0.466 (0.290–0.685)	< 0.001***

*p* value < 0.05*, < 0.01**, < 0.001***

**Table 4:** Univariate and multivariate Cox regression analysis for cancer-specific survival (CSS) in Ewing tumor patients

	Univariate		Multivariate	
Variables	HR (95% CI)	*p* value	HR (95% CI)	*p* value
Age (years)				
≤ 10	Reference	< 0.001***	Reference	< 0.001***
11–20	1.874 (1.381–2.544)	< 0.001***	1.542 (1.132–2.101)	0.006**
21–30	2.407 (1.7063.397)	< 0.001***	1.846 (1.299–2.624)	0.001**
31–49	3.562 (2.444–5.190)	< 0.001***	3.574 (2.429–5.269)	< 0.001***
≥ 50	4.349 (2.657–7.120)	< 0.001***	4.100 (2.479–6.782)	< 0.001***
Gender				
Male	Reference			
Female	0.904 (0.743–1.101)	0.317		
Race				
White	Reference	0.016*	Reference	0.024*
Black	2.046 (1.258–3.327)	0.004**	2.000 (1.215–3.290)	0.006**
Other	1.0223 (0.710–1.475)	0.902	1.018 (0.703–1.475)	0.924
Primary site				
Limb	Reference	< 0.001***	Reference	0.013*
Cranial	1.104 (0.701–1.740)	0.670	1.436 (0.886–2.329)	0.142
Spine	1.421 (0.986–2.049)	0.060	1.394 (0.947–2.052)	0.093
Thoracic	1.049 (0.783–1.407)	0.747	0.897 (0.662–1.215)	0.482
Pelvic	1.817 (1.458–2.265)	< 0.001***	1.408(1.105–1.794)	0.006**
Tumor size (cm)				
< 5	Reference	< 0.001***	Reference	< 0.001***
5–8	1.359 (0.992–1.862)	0.056	1.339 (0.965–1.857)	0.081
> 8	2.017 (1.510–2.695)	< 0.001***	1.877 (1.370–2.572)	< 0.001***
Tumor extent				
Localized	Reference	< 0.001***	Reference	< 0.001***
Regional	1.543 (1.163–2.047)	0.003**	1.400 (1.046–1.874)	0.024*
Metastatic	4.001 (3.014–5.311)	< 0.001***	3.409 (2.520–4.611)	< 0.001***
Cancer-directed surgery				
No	Reference		Reference	
Yes	0.563 (0.465–0.683)	< 0.001***	0.925 (0.740–1.156)	0.491
Radiation				
No	Reference	0.001**	Reference	
Yes	1.366 (1.130–1.652)		1.079 (0.882–1.319)	0.461
Chemotherapy				
No	Reference	< 0.001***	Reference	
Yes	0.431 (0.283–0.656)		0.452 (0.291–0.700)	< 0.001***

*p* value < 0.05*, < 0.01**, < 0.001***

**Fig. 4:**
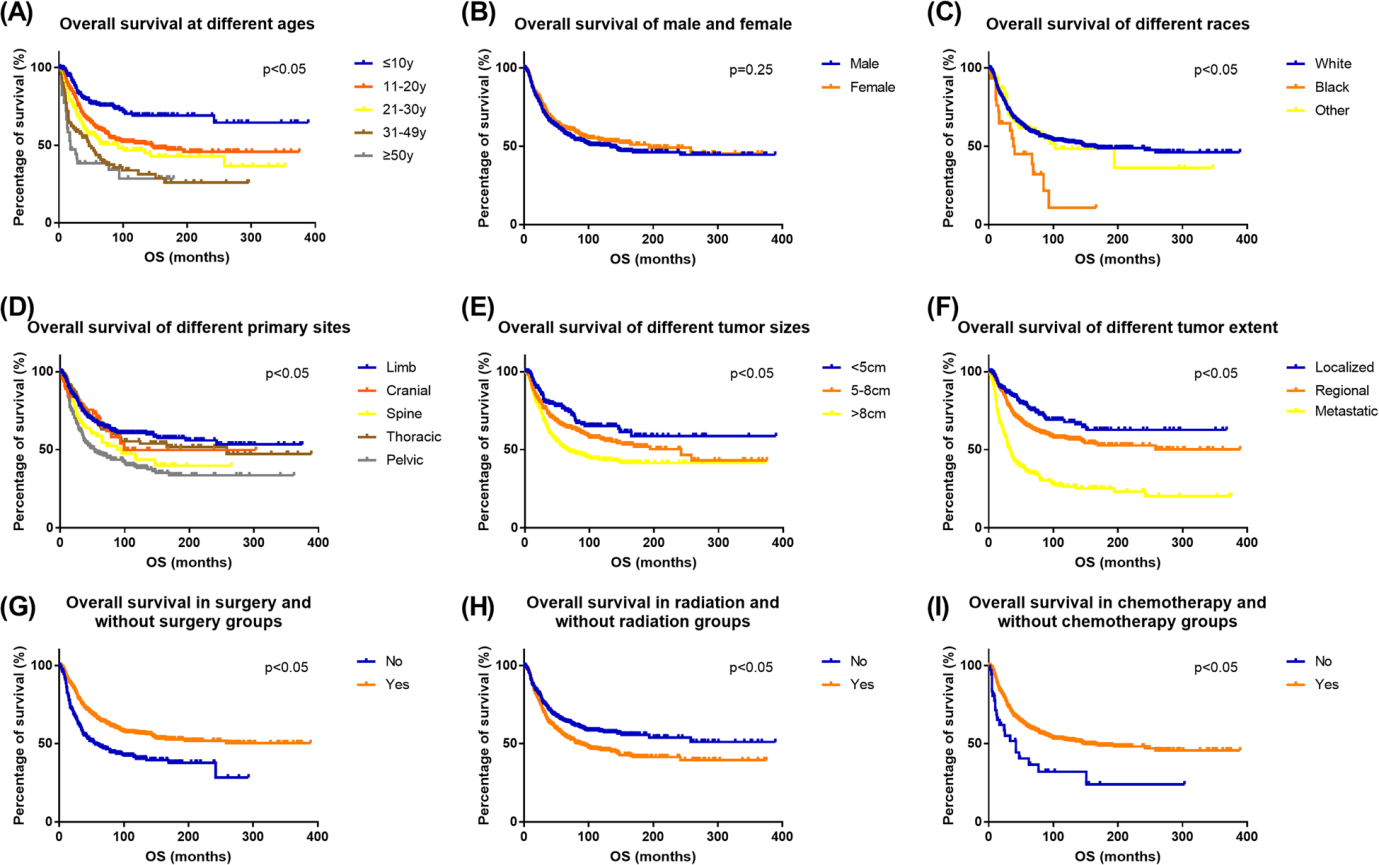
Overall survival (OS) Kaplan-Meier curves for ES patients. **a** age, **b** gender **c** race, **d** primary site, **e** tumor size, **f** tumor extent, **g** cancer-directed surgery, **h** radiation, and **i** chemotherapy

**Fig. 5:**
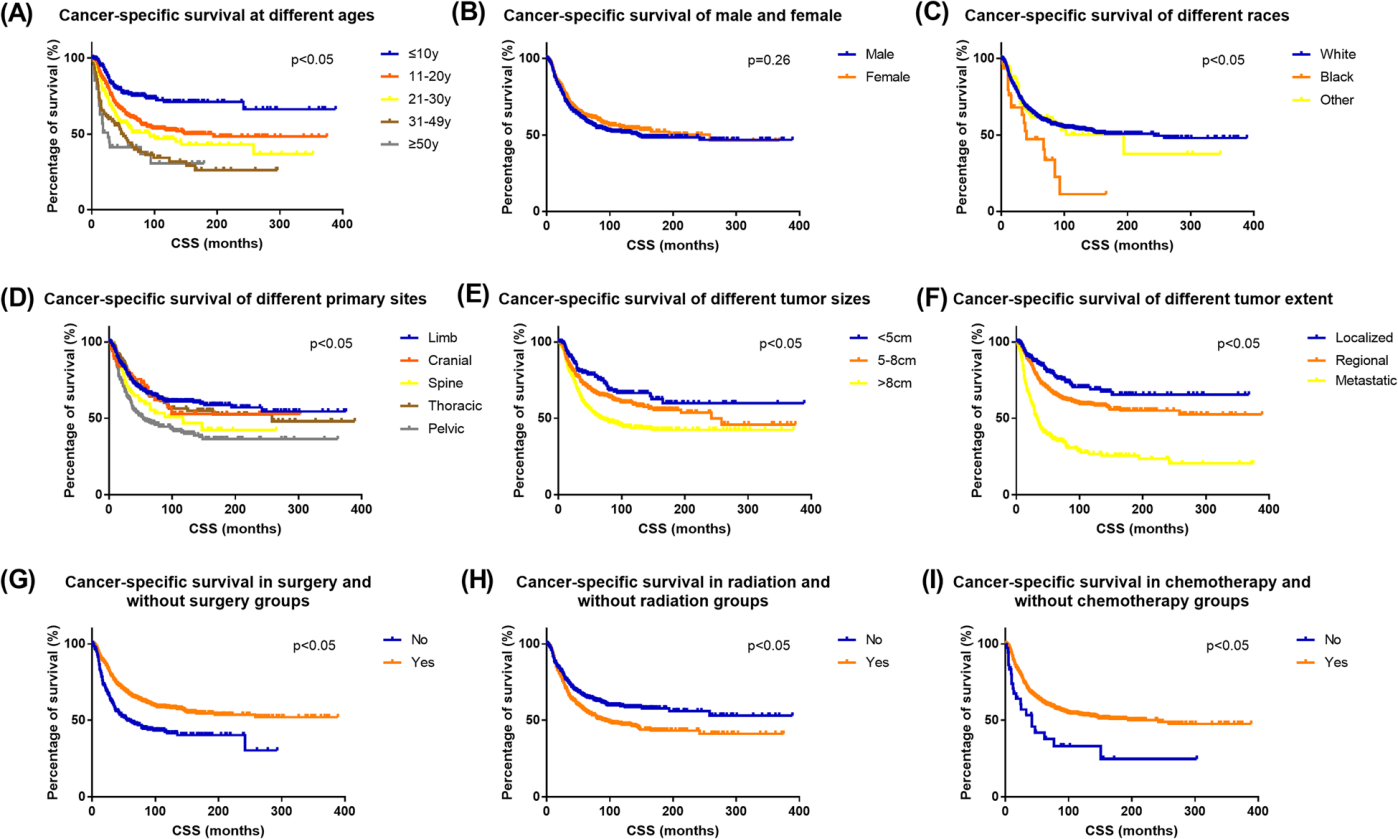
Cancer-specific survival (CSS) Kaplan-Meier curves for ES patients. **a** age, **b** gender, **c** race, **d** primary site, **e** tumor size, **f** tumor extent, **g** cancer-directed surgery, **h** radiation, and **i** chemotherapy

Kaplan-Meier survival curves showed the association between OS and each factor (Fig. 4). CSS results showed consistency with OS (Fig. 5). The curves showed the prognostic factors more visually.

### Nomogram development

**Fig. 6:**
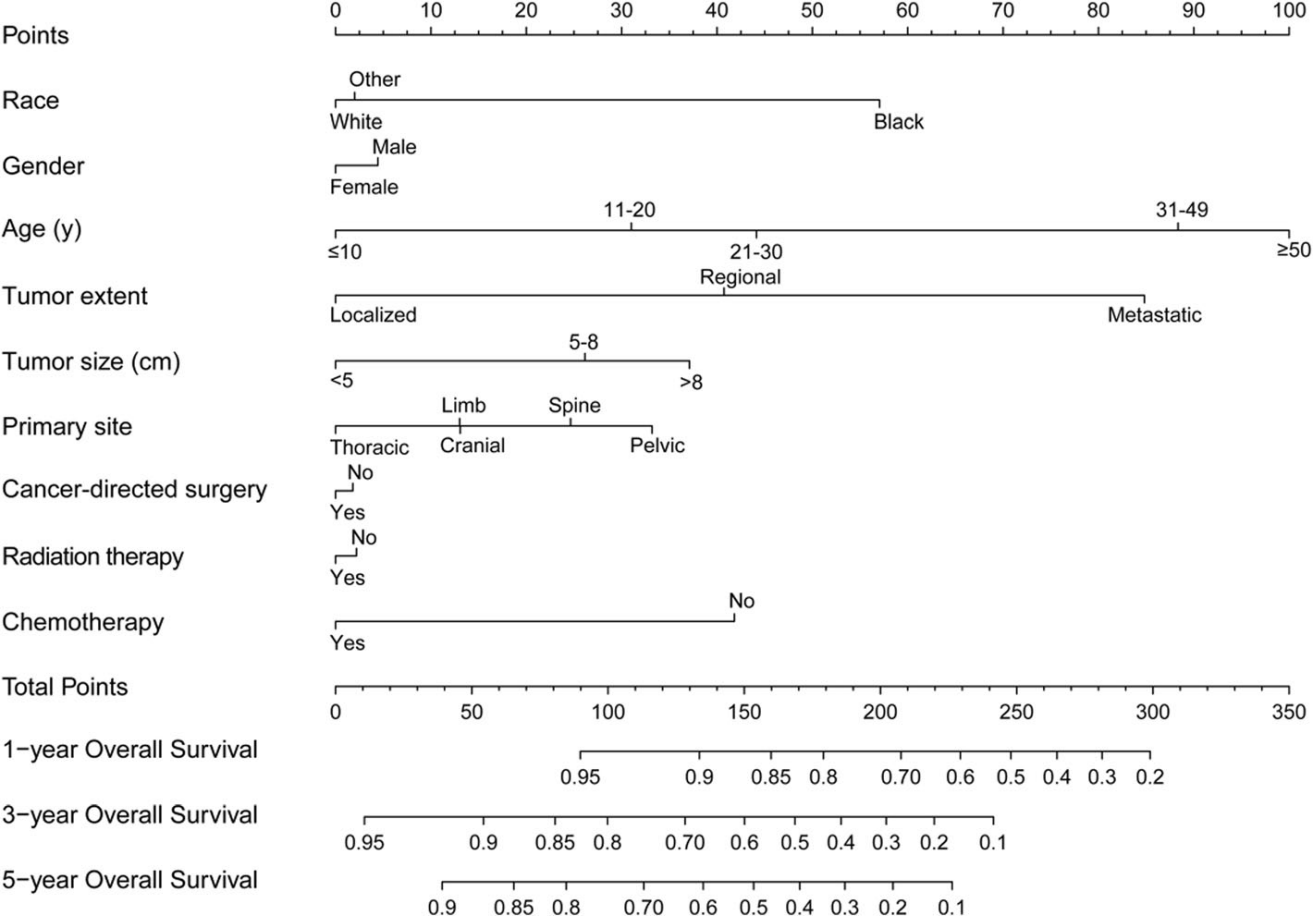
Nomogram predicting the probability of OS

**Fig. 7:**
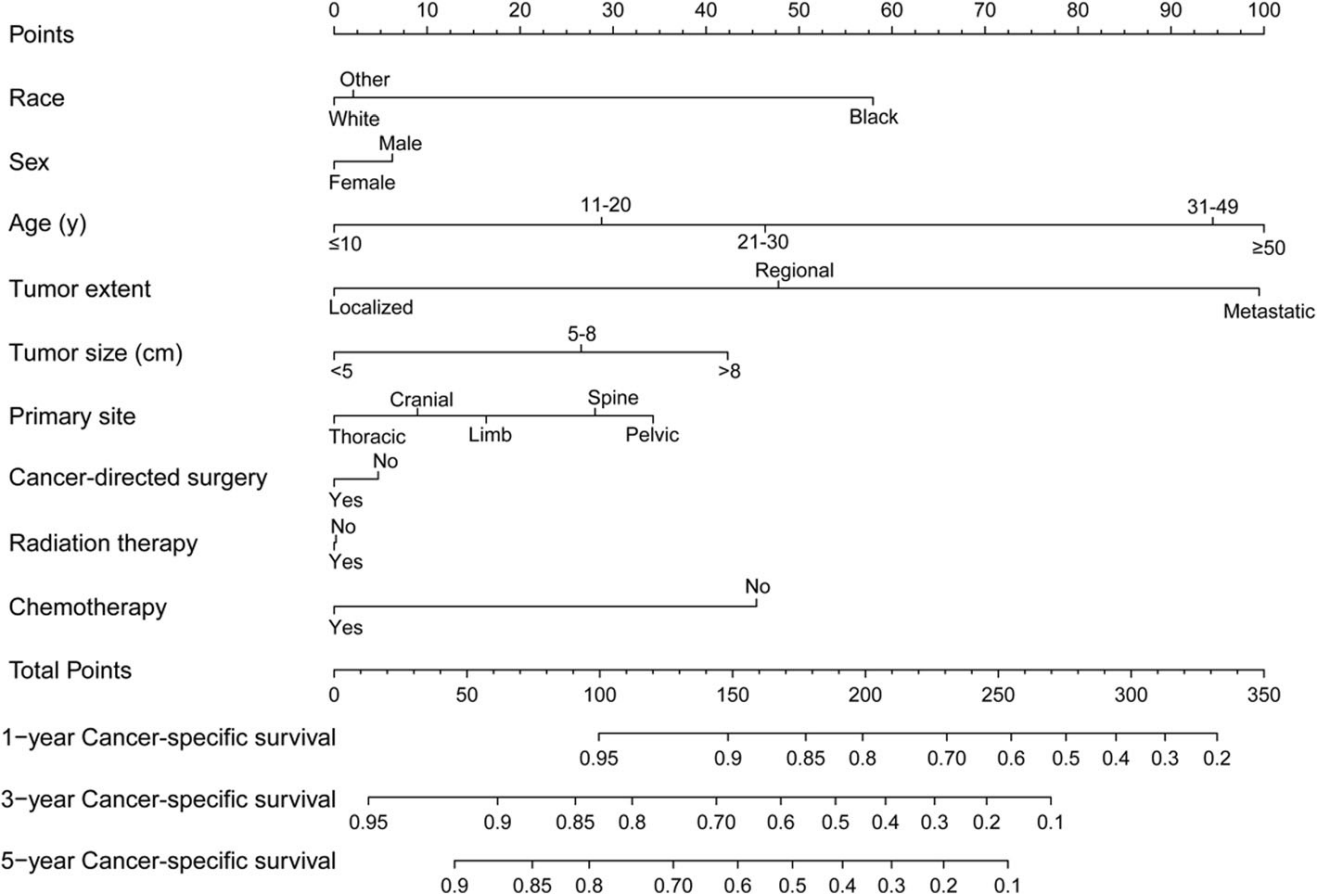
Nomogram predicting the probability of CSS

**Fig. 8:**
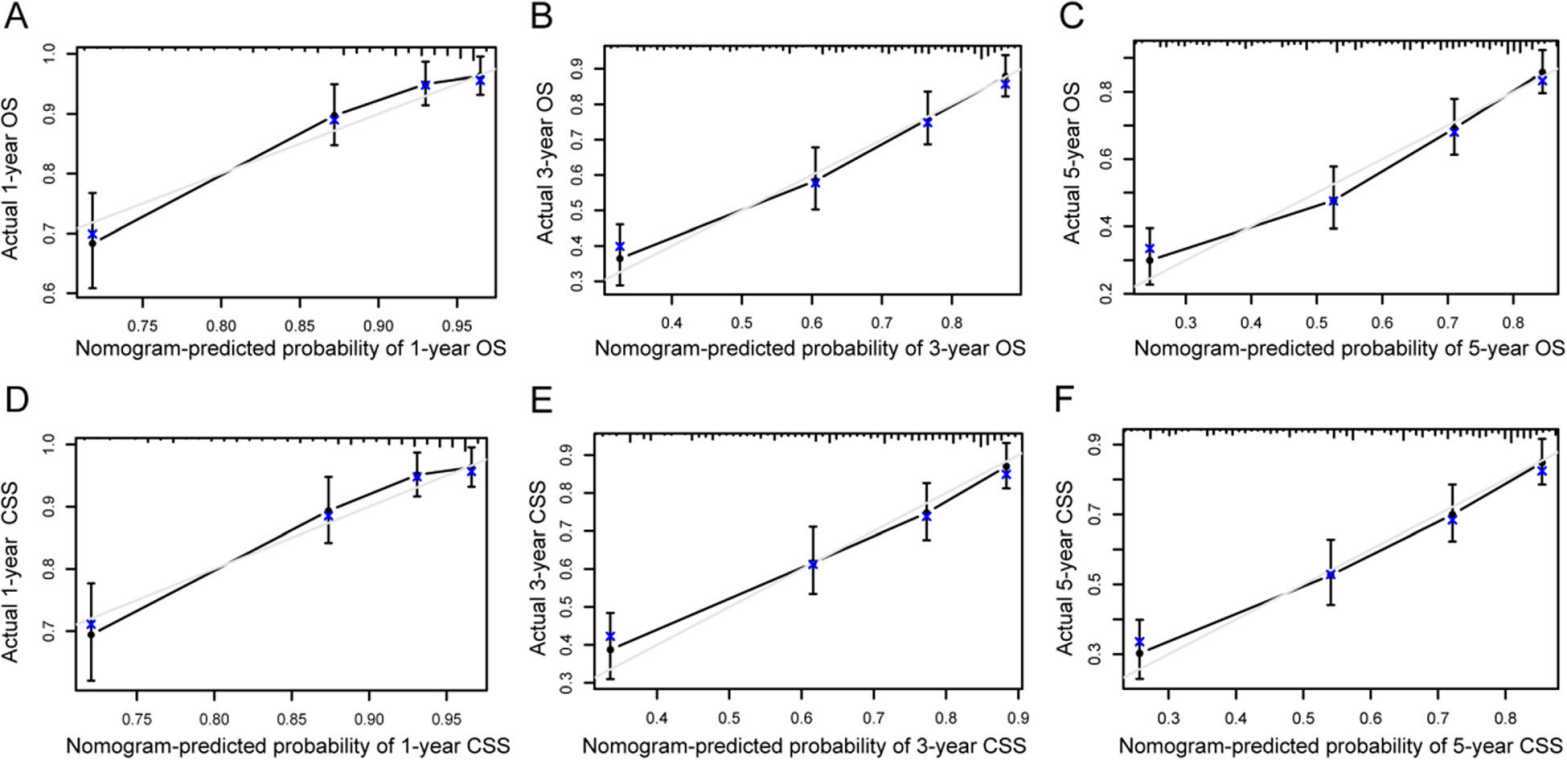
Internal validation calibration plot for nomograms. **a–f** The graphs show the calibration plots for internal validation of **a** 1-year, **b** 3-year, and **c** 5-year overall survival. **d** 1-year, **e** 3-year, and **f** 5-year cancer-specific survival

**Fig. 9:**
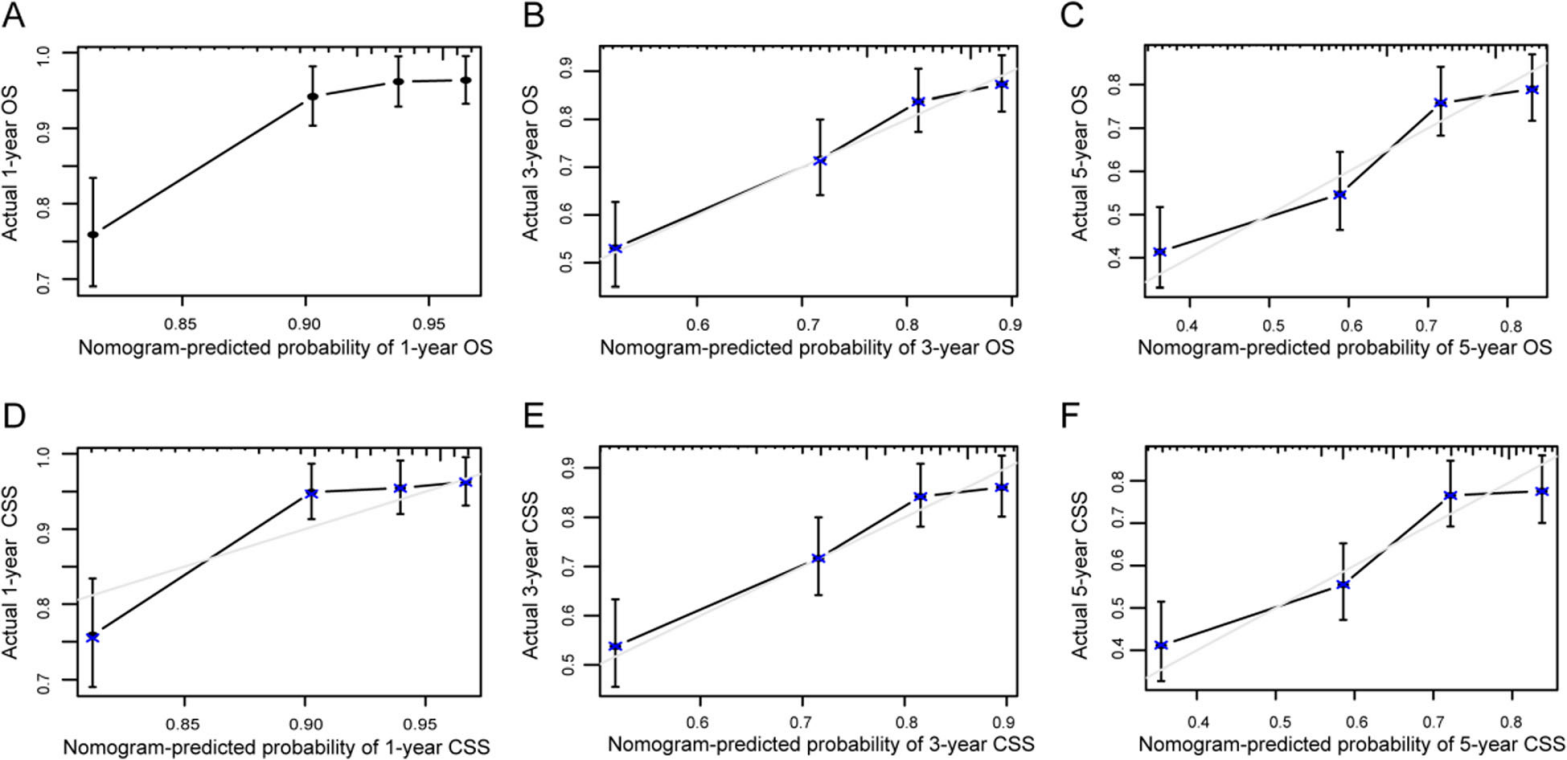
External validation calibration plot for nomograms. **a–f** The graphs show the calibration plots for external validation of **a** 1-year, **b** 3-year, and **c** 5-year overall survival. **d** 1-year, **e** 3-year, and **f** 5-year cancer-specific survival

Nomogram was a good visualization method to quantify the results of Logistic and Cox regression [16]. In our study, we randomly assigned all 1160 patients into training set (570 pts) and validation set (590 pts), as shown in Table 5. Then we incorporated all clinicopathological factors to develop the nomogram to predict the probability of 1-year, 3-year, and 5-year OS (Fig 6) and CSS (Fig 7). Next, we used bootstrap method validation with a resample of 1000 and draw calibration plots to assess the consistency between predicted outcome and observed actual outcome. In the internal validation, C-indices were both 0.724 for OS and CSS (Fig 8). In the external validation, C-indices were 0.688 and 0.689 (Fig. 9). The C-indices were all around 0.7, which showed the good prediction power of the nomograms.

**Table 5:** The baseline characteristics of training and validation set

Characteristic	All patients (*N* = 1160) Number (%)	Traning set (*N* = 570, 49.1%) Number (%)	Validation set (*N* = 590, 50.9%) Number (%)	*p* value
Age (years)				0.349
≤ 10	242 (20.9%)	117 (20.5%)	125 (21.2%)	
11–20	571 (49.2%)	288 (50.5%)	283 (48.0%)	
21–30	206 (17.8%)	105 (18.4%)	101 (17.1%)	
31–49	101 (8.7%)	46 (8.1%)	55 (9.3%)	
≥ 50	40 (3.4%)	14 (2.5%)	26 (4.4%)	
Gender				0.084
Male	733 (63.2%)	346 (60.7%)	387 (65.6%)	
Female	427 (36.8%)	224 (39.3%)	203 (34.4%)	
Race				0.727
White	1041 (89.7%)	511 (89.6%)	530 (89.8%)	
Black	30 (2.6%)	13 (2.3%)	17 (2.9%)	
Other	89 (7.7%)	46 (8.1%)	43 (7.3%)	
Primary site				0.384
Limb	519 (44.7%)	254 (44.6%)	265 (44.9%)	
Cranial	66 (5.7%)	32 (5.6%)	34 (5.8%)	
Spine	88 (7.6%)	37 (6.5%)	51 (8.6%)	
Thoracic	173 (14.9%)	95 (16.7%)	78 (13.2%)	
Pelvic	314 (27.1%)	152 (26.7%)	162 (27.5%)	
Tumor size (cm)				0.296
< 5	221 (19.1%)	119 (20.9%)	102 (17.3%)	
5–8	371 (32.0%)	179 (31.4%)	192 (32.5%)	
> 8	568 (49.0%)	272 (47.7%)	296 (50.2%)	
Tumor extent				0.985
Localized	311 (26.8%)	153 (26.8%)	158 (26.8%)	
Regional	533 (45.9%)	263 (46.1%)	270 (45.8%)	
Metastatic	316 (27.2%)	154 (27.0%)	162 (27.5%)	
Cancer-directed surgery				0.651
No	390 (33.6%)	188 (33.0%)	202 (34.2%)	
Yes	770 (66.4%)	382 (67.0%)	388 (65.8%)	
Radiation				0.636
No	584 (50.3%)	291 (51.1%)	293 (49.7%)	
Yes	576 (49.7%)	279 (48.9%)	297 (50.3%)	
Chemotherapy				0.032*
No	40 (3.4%)	13 (2.3%)	27 (4.6%)	
Yes	1120 (96.6%)	557 (97.7%)	563 (95.4%)	

*p* value < 0.05*

## Discussion

Ewing sarcoma (ES) was a rare bone and joint malignant tumor, and occasionally occurred in the soft tissue and other extra-bone tissues (Extraskeletal E S[17]). After osteosarcoma, ES is the second most common primary bone cancer of children and adolescents, with the median age at diagnosis being 15 years and reported incidence [18]. ES, an extremely aggressive and disseminated tumor, has a high propensity for local recurrences and distant metastases. The most common metastatic sites are the lungs, adjacent, or distant bone/bone marrow. Regional lymph node involvement is rare [19]. Our study showed that tumor size, primary site, and tumor extent were associated with distant metastasis at diagnosis, which was similar to previous studies [4] [7].

ES is a kind of a class of diseases called small round cell malignant tumors. In such diseases, malignant tumor cells presented similar morphology, so it was hard to differentiate histology grade [20] [21]. In our study, we found that the most patients had missed grade data. And in patients who had grade data, most of them were grade III and IV (poorly differentiated of undifferentiated). Considering these situations, so we did not include grade factor in further metastasis and survival analysis, and grade did not show statistically significant results.

Nomogram is a widely used tool nowadays. We use this useful tool to predict the occurrence of a specific event and estimate the prognosis in medicine, particularly in clinical oncology. Besides the ability to generate an individual numerical probability of a clinical event by integrating diverse prognostic and determinant variables, the advantages of visualization and quantification were also practical in clinical practice [22]. Our study developed a nomogram to predict probability of OS and CSS, which could visualize the prognostic risk factors, differentiate high-risk groups, and then predict the prognosis of patients with different clinicopathological characteristics. In clinical practice, it is hard to predict prognosis of a certain patient, and there is no large-size statistical prognosis data of ES patients in China. Furthermore, the most prognosis studies only showed correlations between risk factors and prognosis, but results were not quantified and visualized. Our model integrated multiple clinicopathological factors and provided a prognostic reference for doctors, which could help to predict recurrence and overall survival at the first diagnosis, as well as guide subsequent treatment. If the patient predicted earlier recurrence risk, doctors would consider closer follow-up and surveillance. Although we showed good calibration results, discrimination could also vary if applied to different cohorts, and the nomogram was not always robust, which still needed further validation of other patient cohorts.

With modern multidisciplinary treatment including intensified chemotherapeutic regimens, 5-year overall survival in ES patients was significantly improved to about 70% of patients with localized tumor. However, the prognosis for patients with early relapse or primary distant metastasis remains still dismal [23]. In our clinical practice, chemotherapy was recommended in preoperative, postoperative, and metastatic ES patient, and better controlled metastasis. In our survival analysis, besides basic clinicopathologic factor, such as age, tumor size, primary site, tumor extent, and metastasis, chemotherapy was also associated with OS and CSS. Surgery and radiation were local treatment strategies, and did not show independent prognostic value, and in univariate Cox analysis, radiation was even a poorer prognostic factor. We considered that of surgery and radiation were all local control treatments, and their performances were dependent on the tumor size, primary site, and the tumor extent, which could act as confounder factors. Since not all patients received surgery and radiation, compared with the widespread use of chemotherapy, larger and more widely invaded tumor could probably receive these local treatments, and maybe this patient group had poorer baseline characteristics compared with those who did not receive. As for chemotherapy, it is an independent prognostic factor of both OS and CSS.

For studies of low-incidence diseases like Ewing sarcoma, SEER database brings an incomparable advantage in cohort capacity, yet the corresponding limitations emerge. Previous study showed [22] that different chemotherapy plan, intensity, course, cycle numbers, and treatment response were also important factors associated with long-term prognosis in clinical practice; however, these precise records were difficult to collect in patient cohorts from SEER database. In the data analysis process, we still take chemotherapy as a consideration, but we simplified data as chemotherapy received and non-received. Because in clinical practice, the detailed treatment data is also complex, and we could not take all factors in prognosis analysis. We could only analyze these patients as a common population, and make some adjustments after considering actual situation. For example, if the patient was in good physical condition and showed good response after initial chemotherapy, his prognosis would be expected better than the predicted data based on our nomogram. Furthermore, in small round cell tumor, patients often had genetic and molecular alterations [23], which were also associated with survival and hard to get related data. Additional efforts should be directed at collecting more complete genetic and treatment data, which are also missed in SEER database, and then explore the best personal and precise treatment to improve survival of ES patients. Besides, although the SEER database is advantaged in long time span for diseases of low incidence, there still exist changes in record standard associated with tumor size, staging, and treatment data. Although we have tried to standardized these data in our variate’s classifications, some progresses in treatment patterns such as improvements of surgery, development of radiotherapy technology, and new chemotherapy drugs and regimens were also associated with prognosis. When considering applying our predicted model to clinical practice, it is necessary to do some adjustments based on actual condition.

## Conclusion

Our study included the most clinicopathological factors associated with prognosis of ES patients, and tumor size and primary site were associated with distant metastasis at diagnosis. It was concluded that tumor size and primary site were associated with distant metastasis at diagnosis, and age, tumor size, primary site, tumor extent, and chemotherapy were associated with overall survival and cancer-specific survival. The prognosis predicted model established based on SEER data and nomogram showed good consistency with actual observed outcomes internally and externally, which could help doctors to predict prognosis of a certain patient in actual clinical practice. It would also help to guide treatment, follow-up and elevate treatment precision and individualization.

## Data Availability

The datasets used and/or analyzed during the current study are available either online or from the corresponding author on reasonable request.

## Abbreviations

AUC: Area under curve
CSS: Cancer specific survival
ES: Ewing sarcoma
OS: Overall survival
ROC: Receiver operating characteristic
SEER: Surveillance, epidemiology, and end results
